# Biolubricant basestocks from chemically modified plant oils: ricinoleic acid based-tetraesters

**DOI:** 10.1186/1752-153X-7-128

**Published:** 2013-07-25

**Authors:** Nadia Salih, Jumat Salimon, Emad Yousif, Bashar Mudhaffar Abdullah

**Affiliations:** 1 School of Chemical Sciences and Food Technology, Faculty of Science and Technology, Universiti Kebangsaan Malaysia, 43600 Bangi, Selangor, Malaysia; 2 Department of Chemistry, College of Science, Al-Nahrain University, Baghdad, Iraq

**Keywords:** Ricinoleic acid-based tetraesters, Chemical modification, Biolubricant basestock, Pressurized differential scanning calorimetry (PDSC), Thin-film micro-oxidation (TFMO)

## Abstract

**Background:**

Plant oils have been investigated as a potential source of environmentally favorable biolubricants because of their biodegradability, renewability and excellent lubrication performance. Low oxidation and thermal stability, poor low-temperature properties and a narrow range of available viscosities, however, limit their potential application as industrial lubricants. The inherent problems of plant oils can be improved by attaching functional groups at the sites of unsaturation through chemical modifications. In this article, we will demonstrate how functionalization helps overcome these disadvantages.

**Results:**

In this work, mono-, tri- and tetra-esters have been synthesized, including 10,12-dihydroxy-9-(stearoyloxy)octadecanoic acid **3**; 9,10,12-tris(stearoyloxy)octadecanoic acid **4**; and 18-(4-ethylhexyloxy)-18-oxooctadecane-7,9,10-triyl tristearate **5**. Pour-point and cloud-point measurements have shown that these derivatives have improved low-temperature properties as compared to the precursor. The tetra ester compound, 18-(4-ethylhexyloxy)-18-oxooctadecane-7,9,10-triyl tristearate **5**, had the lowest pour point (PP) (−44.37°C) and the lowest cloud point (CP) (−41.25°C). This derivatization also improved the compound’s thermo-oxidative stability, measured using pressurized differential scanning calorimetry (PDSC) and thin-film micro-oxidation (TFMO) testing. 18-(4-Ethylhexyloxy)-18-oxooctadecane-7,9,10-triyl tristearate **5** also had the highest onset temperature (OT) (282.10°C) and the lowest volatile loss and insoluble deposit (37.39% and 50.87%, respectively). Furthermore, the compounds’ tribological behaviors were evaluated using the four-ball method. 18-(4-Ethylhexyloxy)-18-oxooctadecane-7,9,10-triyl tristearate **5** also had the lowest coefficient of friction (μ) (0.44).

**Conclusions:**

The results showed that, in general, these derivatives have good anti-wear and friction-reducing properties at relatively low concentrations under all of the test loads. Overall, the data indicates that these derivatives have significant potential to be used as biolubricant basestocks.

## Background

Environmental concerns over the use of petroleum-based products in various industries, such as forestry, farming, mining, and boating have led to increased interest in the use of environmentally friendly fluids [1]. Growing environmental concerns are providing the impetus for increasing the demand and usage of plant oils in lubricants for many applications [2,3]. Approximately 10–15 million tons of petroleum-based oleochemicals enterthe biosphere every year, and approximately 40% come from spills, industrial and municipal waste, urban runoff, refinery processes, and condensation from marine engine exhaust. Oleochemical pollutants are derived from the food industry, petroleum products, and by products, such as lubricating hydraulic and cutting oils [4]. Renewable raw materials are going to play a noteworthy role in the development of sustainable green chemistry, and plant oils have gained increasing attention because of their renewability. They offer a large number of possibilities for applications that can rarely be achieved by petrochemistry. Oils and fats from plant oils share the greatest proportion of the current consumption of renewable raw materials in the chemical industry [5]. A steady increase in the use of eco-friendly consumer products such as lubricants has occurred as a result of strict government regulation and increased public awareness for a pollution-free environment [6]. There are wide ranges of lubricant base oils in current use, including mineral oils, synthetic oils, re-refined oils, and plant oils. Among these, mineral oils are the most commonly used. They consist predominantly of hydrocarbons but also contain some sulfur and nitrogen compounds with traces of a number of metals.

The beneficial aspects of biolubricants from renewable sources have long been recognized. In general, biolubricants have very low or almost negligible aquatic toxicity and are, in most cases, readily biodegradable. Biodegradability mainly depends on the chemical structure. Frequently, higher chemical stability results in reduced degradation rates [7,8]. Plant oil lubricants also acquire most of the properties required for lubricants such as high viscosity indices (VI) because of their high molecular weights, low volatility (they have an approximately 20% lower rate of evaporation than mineral-oil-based fluids) and good lubricity because their ester bonds enable the oil molecules to cling to metal surfaces *via* physical bonding and provide better boundary lubricity than nonpolar petroleum-based mineral oil. Additionally, bio-based oils have superior compatibility with additive molecules [9]. However, typical plant oils, such as soybean or rapeseed oils, cannot fully meet the performance criteria for most lubricants. High levels of unsaturated fatty acids, such as oleic, linoleic, and linolenic acids, are present in plant oils and maintain the fluidity of cell membranes. However, the presence of bis-allylic protons in these oils makes them susceptible to oxidation. Increasing the degree of saturation of the oil usually results in poor low-temperature properties [10]. Most plant oils crystallize when the temperature is below refrigeration temperature. The solidification points of common plant oils are summarized in Table 1[11]. Other shortcomings of plant oils include deposit-forming tendencies, and low hydrolytic stabilities [9].

**Table 1:** Solidification points of common plant oils

**Name**	Solidification point* (°C)
Castor oil	–17 to –18
Corn oil	–10 to –20
Cottonseed oil	12 to –13
Linseed oil	–19 to –27
Palm oil	35 to 42
Palm kernel oil	27
Peanut oil	3
Rapeseed	–10
Safflower oil	–13 to –18
Sesame oil	–4 to –6
Soybean oil	–10 to –16
Sunflower oil	–17

*The solidification points depend on varieties.

The most serious disadvantage of the usage of plant oils in biolubricants is their poor thermo-oxidative stability [12]. Plant oil oxidizes similarly to hydrocarbon mineral oil, following the same free radical oxidation mechanism but at a faster rate. The faster oxidation of plant oils is due to their unsaturated fatty acids (bis-allylic hydrogens in linoleic and linolenic fatty acids are susceptible to free radical attacks), peroxide formation and the production of polar oxidation products [13]. Different modern technological approaches have been adopted to solve the problems associated with the application of plant oils in biolubricants. However, low resistance to oxidative degradation still remains the major drawback to the application of plant oil in biolubricants [14]. The physical and chemical properties of plant oils are determined by their fatty acid (FA) profiles. Table 2 shows typical fatty acid contents of some plant oils that are being investigated as potential basestocks for industrial applications [11]. High unsaturation in the molecule increases the rate of oxidation, resulting in polymerization and an increase in viscosity. However, high saturation increases the melting point of the oil [15]. Therefore, suitable adjustments between the low-temperature properties and oxidative stability must be made when selecting a plant oil basestock for particular industrial applications.

**Table 2:** Fatty acid compositions (%) of plant oils by GC analysis

**Plant oil**	**Palmitic**	**Stearic**	**Oleic**	**Linoleic**	**Linolenic**
Safflower oil	6	4	20	70	-
High-oleic safflower oil	5	3	80	12	-
High-linoleic safflower oil	3	2	10	85	-
Sunflower oil	6	-	35	55	4
High-oleic sunflower oil	5	-	90	5	-
Soybean oil	16	-	23	53	8
Corn oil	7	-	47	42	4
Cotton seed oil	22	-	25	50	3

The performance limitations of plant oil basestocks can be overcome through chemical modification. Current research efforts are directed toward improving the thermal and low-temperature stability of plant oils by chemical modification [16]. The molecular structures of plant oils have some potential sites for chemical modification, such as at double bonds, and the conversion of C = C bonds to oxirane rings *via* epoxidation constitutes a promising approach to obtain valuable commercial products from renewable raw materials. Epoxidation has received special attention because it opens up a wide range of feasible reactions that can be carried out under moderate reaction conditions because of the high reactivity of the oxirane ring [17-19].

The goal of this work is to continue developing a chemical modification method for the preparation of biolubricant basestocks from ricinoleic acid [20]. The synthesis, characterization, and utilization of head-group manipulation, together with a branching strategy to improve the physicochemical and tribological properties of the ricinoleic-acid-based-tetraesters, are discussed within this contribution.

## Results and discussion

### Synthesis

**Figure 1:**
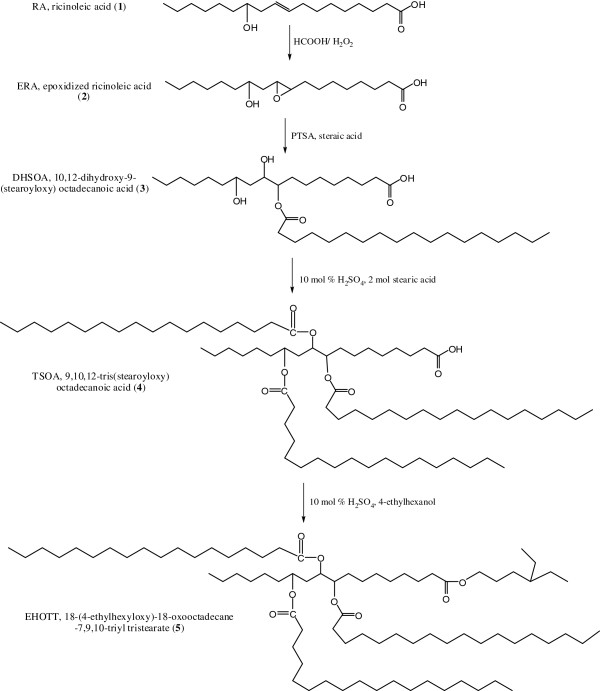
Reaction scheme for the formation of tetraesters.

Due to the increasing market relevance of environmentally labeled products, the ecological properties of plant oil biolubricants have been intensively studied over the last few years [18,19]. In general, their aquatic toxicity is very low or almost negligible, and they are readily biodegradable in most cases. Their origin from renewable resources results in lower net CO_2_-emissions (“global warming”) compared to petrochemical products. Few lubricants have such positive ecological profiles. Thus, most of lubricants are classified in the most favorable water hazard class. The favorable ecological properties of plant oils, together with their well-established technical performance, make them suitable base oils for the development of high-performance “green” oil and biolubricant products [21]. However, typical plant oils cannot fully meet the performance criteria for most lubricants. One approach to eliminate the negative properties of plant oils and to improve their performance is through structural chemical modifications. In this work, careful ring opening of epoxidized ricinoleic acid **1** was carried out to obtain 10,12-dihydroxy-9-(stearoyloxy) octadecanoic acid **3**. Then, esterification of these products was carried out using stearic acid and sulfuric acid as the catalyst to yield 9,10,12-tris(stearoyloxy) octadecanoic acid **4**. This triester compound was used as a precursor for the synthesis of modified tetraester-derivative **5** by an esterification reaction with 4-ethylhexanol (Figure 1). This method is effective for introducing branching on the fatty acid chains of plant oils. The straightforward epoxidation of ricinoleic acid **1** was closely monitored (27°C and 5 hrs) to avoid the synthesis of the undesired 9,10,12-trihydroxyoctadecanoate, which will form if the reaction temperature is elevated or if the reaction is allowed to progress for too long. The removal of unsaturated moieties in the ricinoleic acid through conversion into epoxy-groups improves the oxidative stability of the compound. It has already been established that the presence of multiple double bonds in plant oil fatty acid chains accelerates oxidative degradation [22-24]. Furthermore, a suitable approach to improve the low-temperature properties is to attach branching sites at the epoxy carbons.

### Characterization

**Figure 2:**
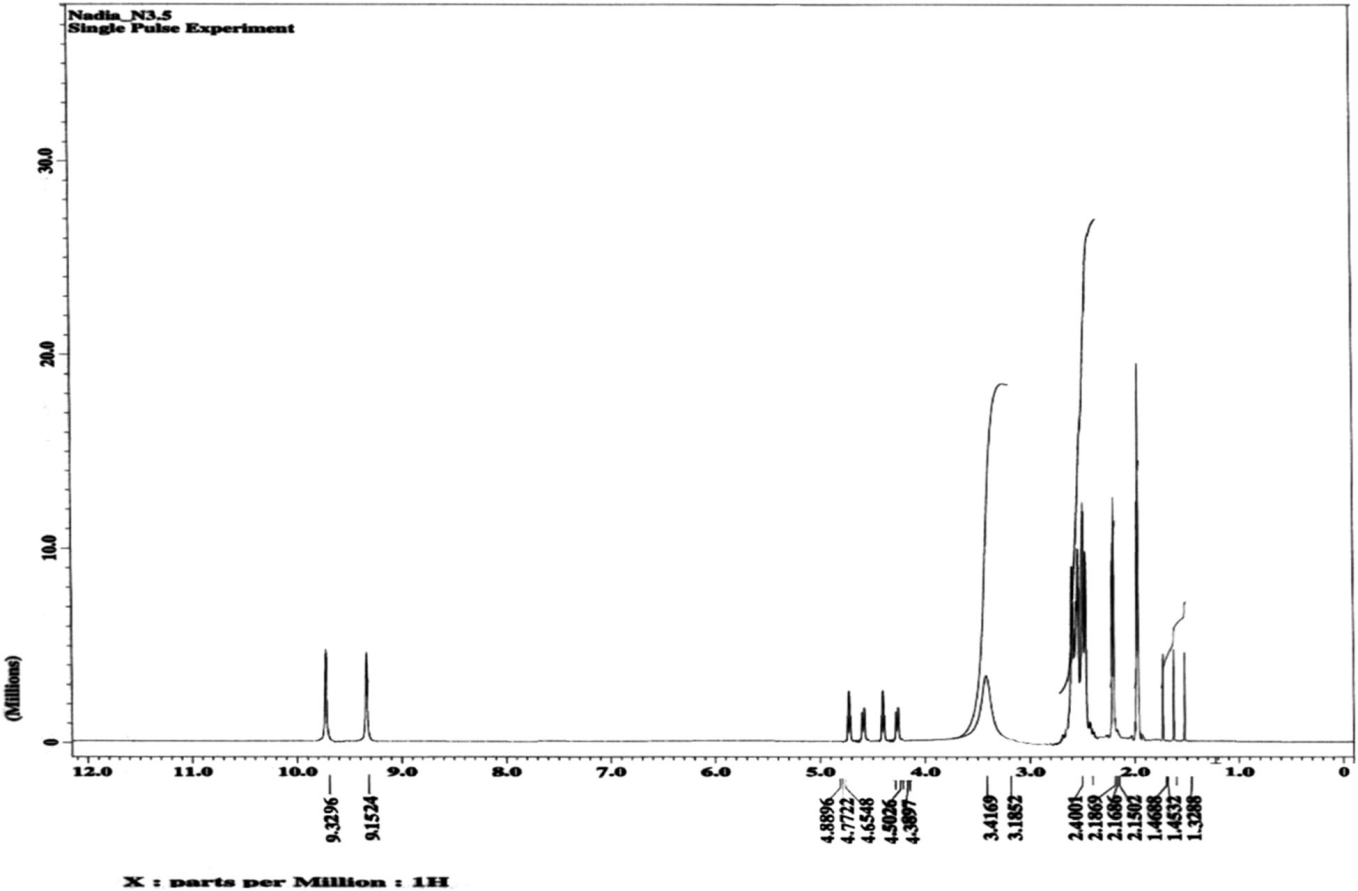
^**1**^**H NMR spectrum for epoxidized ricinoleic acid (ERA) (2).**

**Figure 3:**
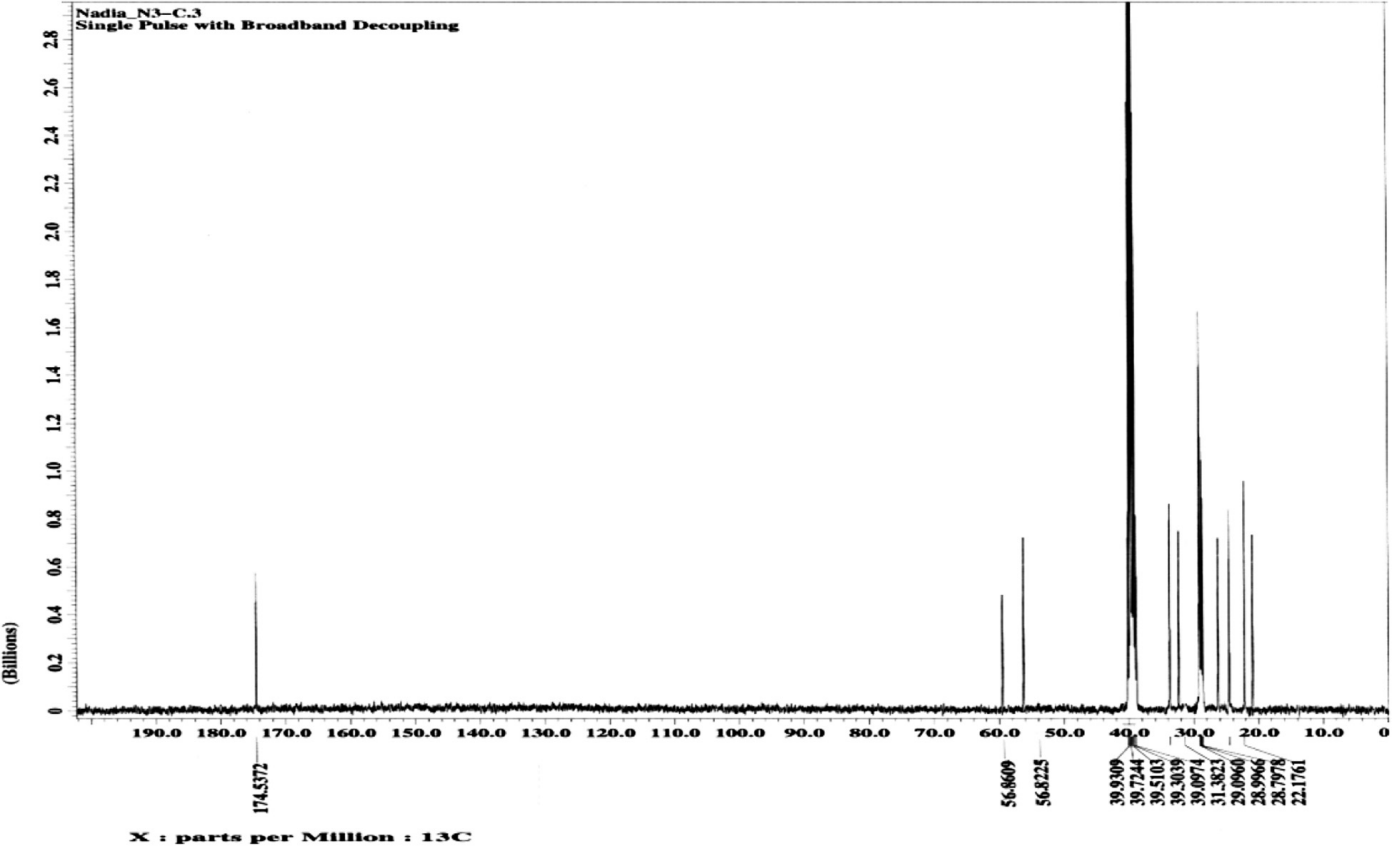
^**13**^**C NMR spectrum for epoxidized ricinoleic acid (ERA) (2).**

All of the synthesized compounds were characterized *via*^1^H, ^13^C NMR and FTIR spectroscopy. Significant signals found in the ^1^H spectrum of the epoxidized ricinoleic acid **2** at ~2.2 and 2.4 ppm correspond to protons on the quaternary carbons of the oxirane ring (Figure 2), whereas a doublet in the ^13^C spectrum at ~56.82 and 56.86 ppm corresponds to the carbons of the oxirane ring (Figure 3). Furthermore, the ^1^H spectrum of epoxidized ricinoleic acid **2** exhibited singlet signals at ~ 9.15 and ~9.32 ppm, which represent the protons of the –O**H** groups [25].

**Figure 4:**
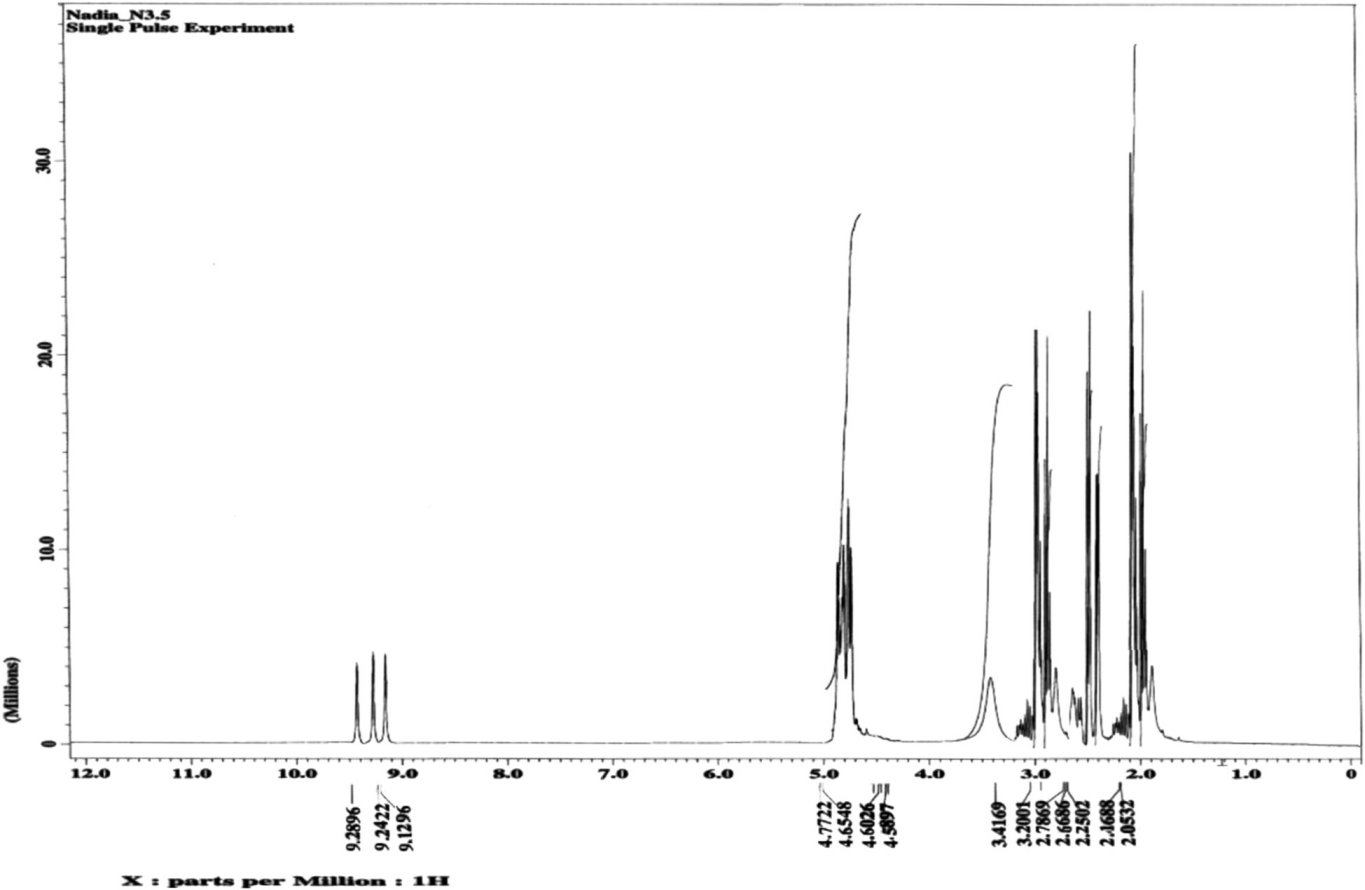
^**1**^**H NMR spectrum for 10,12-dihydroxy-9-(stearoyloxy) octadecanoic acid (DHSOA) (3).**

**Figure 5:**
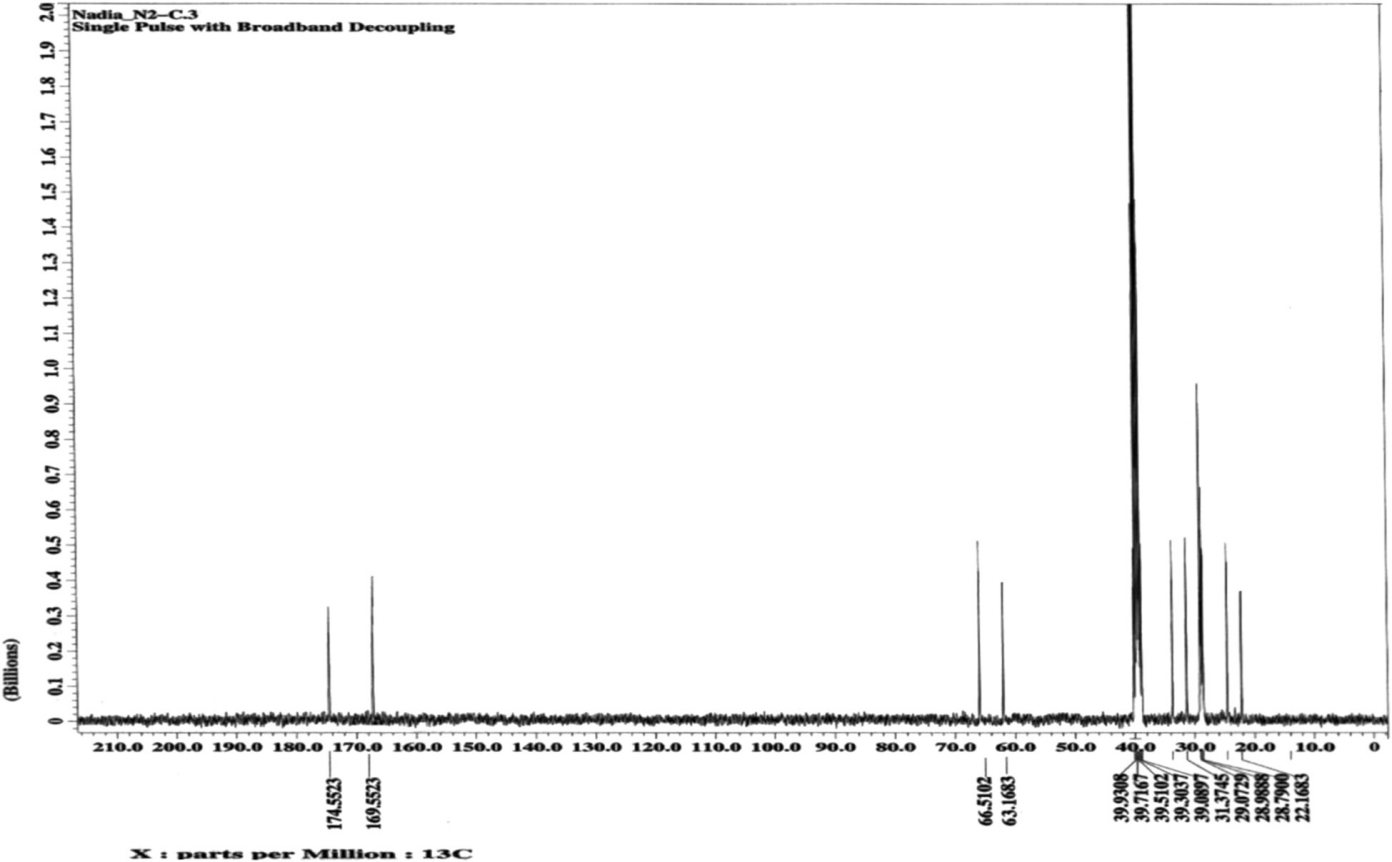
^**13**^**C NMR spectrum for 10,12-dihydroxy-9-(stearoyloxy) octadecanoic acid (DHSOA) (3).**

A singlet at ~9.12-9.29 ppm represents the –O**H** protons, and the bands at ~2.03-3.64 ppm correspond to –C**H**_2_– groups in the ^1^H spectrum of 10,12-dihydroxy-9-(stearoyloxy) octadecanoic acid derivative **3** (Figure 4). The ^13^C NMR signals of 10,12-dihydroxy-9-(stearoyloxy) octadecanoic acid derivative **3** are in agreement with the proposed structure (Figure 5).

The ^1^H spectrum of 9,10,12-tris(stearoyloxy) octadecanoic acid **4** consists of multiplet signals at ~9.18-9.35 ppm due to the –O**H** protons and at ~1.42-3.24 ppm due the –C**H**_2_–, -C**H**(OH) and -C**H**(OCOR) protons. Furthermore, the signals at ~173.1-176.7 ppm in the ^13^C NMR spectrum are attributed to the ester carbonyl groups [23]. These signals are in agreement with the proposed structures. The spectrum of tetraester 18-(4-ethylhexyloxy)-18-oxooctadecane-7,9,10-triyl tristearate **5** consists of signals of low intensity at approximately 9.22-9.40 ppm and 2.10-3.65 ppm. Broad peaks at 1.41-1.77 ppm represent the CH_2_ groups’ hydrogen.

**Figure 6:**
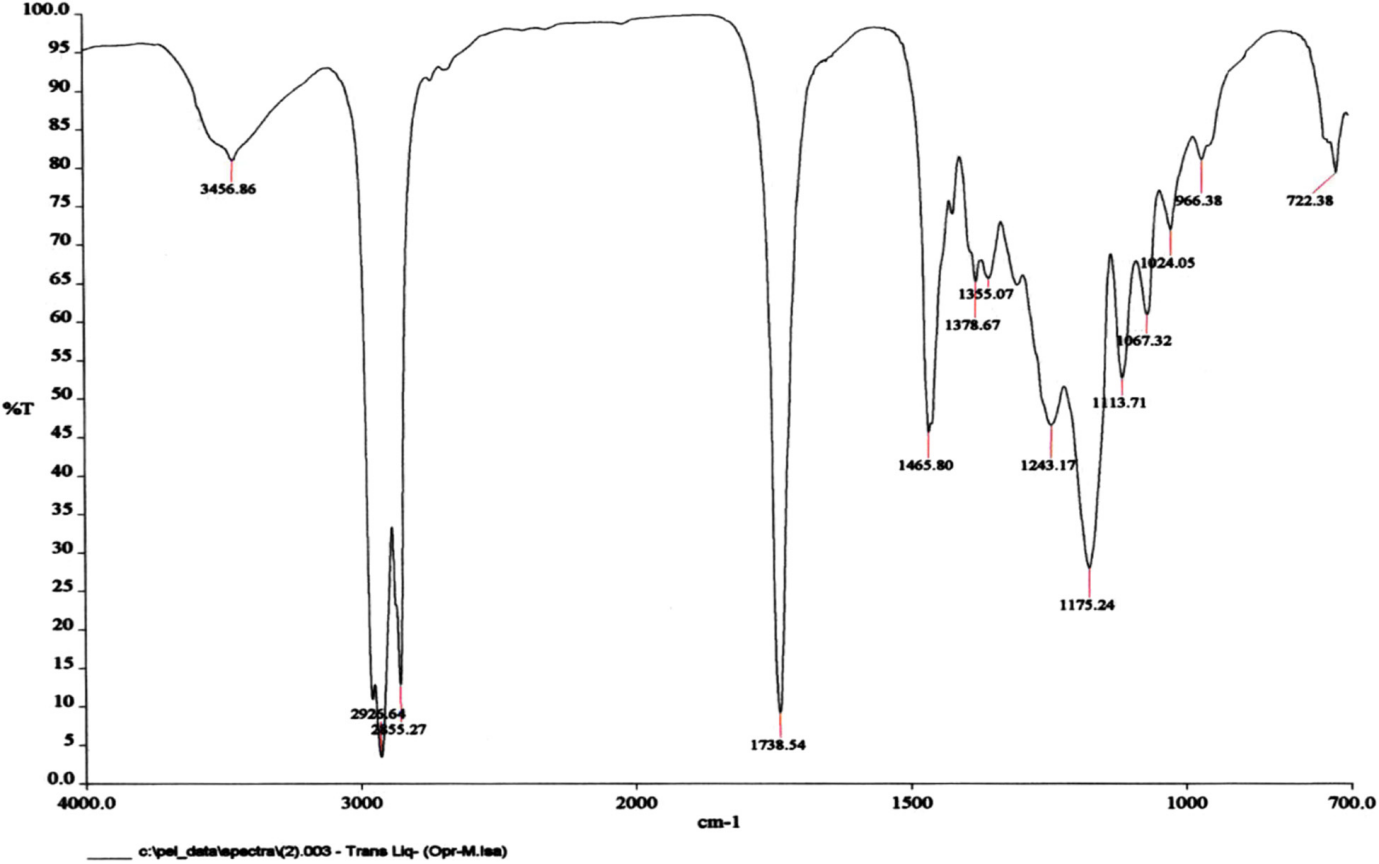
FTIR spectrum for 10,12-dihydroxy-9-(stearoyloxy) octadecanoic acid (DHSOA) (3).

The structures of the ester functional groups were also confirmed *via* IR spectral analysis (Figure 6). Bands representing the ester C = O group (~1740 cm^-1^), the CH_3_ group (~1376 cm^-1^), the OH group (~3478-3443 cm^-1^) and the C-O-C functionality (~1000-1100 cm^-1^) are clearly visible in the spectra [26].

### Low-temperature properties and the viscosity index

Plant oils when subjected to low-temperature environment undergo solidification through crystallization, therefore posing a major hurdle to the use of plant oils in industrial applications. The relatively poor low-temperature flow properties of plant oils derive from the appearance of waxy crystals that rapidly agglomerate, resulting in solidification of the oil. Plant oil is a complex molecular system and, therefore, the transition from the liquid to solid state does not occur at a particular temperature but rather over a wide temperature range involving several polymorphic forms (α, β′, β), contributing to the appearance of wax and the crystallization process. This deposition of waxy materials from the oils results in a rapid increase in viscosity, leading to poor pumpability, lubrication, and rheological behavior [27]. The pour point (PP) and cloud point (CP) of a biolubricant are good indicators of its low-temperature fluidity. The attachment of an ester side chain of optimum length at the 9 or 10 position of the fatty acid chain improved the pour point significantly [28].

Therefore, compounds **2–5** were screened for cold-flow performance through the determination of their cloud and pour points. While epoxidized ricinoleic acid **2** had a pour point of 9.10°C and a cloud point of 12.31°C, all of the synthesized esters **3**–**5** had much better pour points. The tetraester product, 18-(4-ethylhexyloxy)-18-oxooctadecane-7,9,10-triyl tristearate **5** showed the most effective decrease in the pour point and cloud point, −44 and −41°C, respectively. It can be assumed that the presence of a large branching group at the mid and end points of a fatty acid chain creates a steric barrier around the individual molecule and inhibits crystallization, the result of which is lowering of the pour and cloud points (Table 3).

**Table 3:** The physicochemical properties of the samples

**Samples**	**Density (g/cm**^**3**^**)**	**Volatility at 120°C (%)**	**Pour point/ °C**	**Cloud point/ °C**	**Viscosity index (VI)**
**2**, ERA	0.734	1.77	9	12	67
**3**, OHSOA	0.867	1.12	−20	−18	163
**4**, TSOA	1.326	0.89	−32	−29	193
**5**, EHOTT	1.560	0.56	−44	−41	257

*ERA* epoxidized ricinoleic acid, *DHSOA* 10,12-dihydroxy-9-(stearoyloxy) octadecanoic acid, *TSOA* 9,10,12-tris(stearoyloxy) octadecanoic acid, *EHOTT* 18-(4-ethylhexyloxy)-18-oxooctadecane-7,9,10-triyl tristearate.

A lubricant is usually used as a thin film between devices to protect them against the asperities of the devices. The contact pressure between the devices from sliding is usually sufficient to cause surface wear and damage without a protecting substance. In general, a lubricant can reduce friction and minimize wear between the components of mechanical devices. Moreover, a lubricant can also be used as a bridge for heat transfer to remove heat from the device during operation. The viscosity of the lubricant is a very important property in choosing a lubricant for hydrodynamic lubrication. A high viscosity of the lubricant also requires a large force against its own intermolecular forces in sliding between devices. In contrast, if the viscosity of the lubricant is too low, it will cause the surfaces between the devices to be rubbed directly and further damage the devices [29]. The results (Table 3) indicate that the viscosity index increases with an increasing number of substituents at the mid- and end-points, which leads to an overall increase in the molecular weights of the products, resulting from an increased number of ester functionalities.

### Thermo-oxidative stability

Fundamental knowledge of the oxidative properties of lubricants is necessary to predict the long-term thermal stability of these fluids, which is a critically important lubricant property. Oxidation properties evaluated experimentally are often used to predict the actual lubricant service life in high temperature and other extreme applications. The more resistant a lubricant is to oxidation, the lower the tendency it has to form deposits, sludge, corrosive byproducts in grease, engine oil and industrial oil applications and also the more resistant to undesirable increase in viscosity during use.

The triacylglycerol structure forms the backbone of most available plant oils, and these are associated with different fatty acid chains. Therefore, a complex association of different fatty acid molecules attached to a single triglycerol structure constitutes a plant oil matrix. Unsaturation in the triacylglycerol molecule from C = C in the oleic, linoleic, and linolenic acid moieties functions as the active sites for various oxidation reactions. Saturated fatty acids have relatively high oxidation stability [15], which decreases with increasing unsaturation in the molecule. Oxidation of hydrocarbons usually takes place through a radical initiated chain mechanism [30] involving initiation (RH → R^•^, R^•^ + O_2_ → RO_2_^•^), propagation (RO_2_^•^ + RH → RO_2_H + R^•^, R^•^ + O_2_ → RO_2_^•^), branching (RO_2_H → RO^•^ + ^•^OH, RO^•^ + RH + O_2_ → ROH + RO_2_^•^, ^•^OH + RH + O_2_ → H_2_O + RO_2_^•^), chain-stopping inhibition (InH + RO_2_^•^ → In^•^ + RO_2_H) and peroxide decomposition (RO_2_H → inert products). The free radicals generated during the initiation stage react with O_2_ to form peroxy free radicals and hydroperoxides. During this period, O_2_ is consumed in a zero-order process [31], apparently leading to inter-mediates that are not well characterized, prior to the formation of peroxides. The latter undergoes further reactions to form alcohols, ketones, aldehydes, and carboxylic acids [32], leading to rancidity and toxicity [33], thereby accelerating the oil degradation process [34]. These compounds have molecular weights that are similar to plant oils and therefore remain in solution. As the oxidation proceeds, the oxygenated compounds polymerize to form viscous material that, at a particular point, becomes insoluble oil, leading thickening and deposits. The extent of oxidation and formation of oxidation products is further complicated by the amount of unsaturation, structural differences in the various triacylglycerol molecules, and the presence of antioxidants. All of these factors, together or individually, can change the specific compounds formed and the rates of their formation [35]. In addition to unsaturation in the molecule, oxidative degradation and the kinetics of oxidation are also influenced by the methylene chain length and bis-allylic methylene groups, among other factors. The cumulative effect of various structural parameters in the triacylglycerol molecule makes oxidation a highly complex process and no simple kinetic model alone would be adequate for such systems.

Thermal analyses such as pressure differential scanning calorimetry (PDSC) methods, are popular for the determination of the oxidative stabilities of plant oils [36-38]. In this work, pressure differential scanning calorimetry analysis was carried out using a programmable heating rate mode with a constant flow of dry O_2_ inside the pressure chamber to determine the onset temperature (OT) and the signal maximum temperature (SMT). The oxidation onset temperature is a relative measure of the degree of oxidative stability in a material evaluated for a given heating rate and oxidation environment. For example, in oxygen, a higher OT value indicates a more oxidatively stable material. The signal maximum temperature is the temperature at which maximum heat output is observed in the sample during oxidative degradation. A higher SMT value does not necessarily correlate with improved oxidative stability.

The results in Table 4 indicate that a general improvement in the oxidative stability was observed in synthesized compounds **3**–**5**. The oxidative stability increases with an increasing number of substituents (mid- and end-chain esters) and a decreasing number of hydroxyl groups in the synthesized product structures. This phenomenon may occur because the presence of hydroxyl groups increases the possibility of the formation of free radicals, which will facilitate the oxidative degradation mechanism.

**Table 4:** The PDSC data of the samples showing the onset of oxidation and the signal maximum temperatures

**Samples**	**Onset temperature (OT)/ °C**	**Signal maximum temperature (SMT)/ °C**
**2**, ERA	60.23	228.98
**3**, OHSOA	190.78	189.97
**4**, TSOA	224.45	231.21
**5**, EHOTT	282.10	176.59

*ERA* epoxidized ricinoleic acid, *DHSOA* 10,12-dihydroxy-9-(stearoyloxy) octadecanoic acid, *TSOA* 9,10,12-tris(stearoyloxy) octadecanoic acid, *EHOTT* 18-(4-ethylhexyloxy)-18-oxooctadecane-7,9,10-triyl tristearate.

Another thermal-oxidative stability test was performed using thin-film micro-oxidation (TFMO) to study the volatility and the depository tendencies of the synthesized ester products. The test is especially effective when thermally induced volatility is low and when insoluble deposit formation through polymerization is to be considered [39]. In this work, a thin-film micro-oxidation test was carried out the synthesized products **2**–**5**; the data from these samples are presented in Table 5. One can observe that the losses due to high-temperature volatility and insoluble deposits decreased gradually as the number of substituents in the mid-chain ester and end-chain ester groups increased. These results are in agreement with those obtained *via* pressure differential scanning calorimetry (Table 4).

**Table 5:** The thin-film micro-oxidation test data of the samples at 175°C

**Samples**	**Volatile loss (%)**	**Insoluble deposit (%)**
**2**, ERA	65.87	73.78
**3**, OHSOA	55.16	64.32
**4**, TSOA	48.08	57.56
**5**, EHOTT	37.39	50.87

*ERA* epoxidized ricinoleic acid, *DHSOA* 10,12-dihydroxy-9-(stearoyloxy) octadecanoic acid, *TSOA* 9,10,12-tris(stearoyloxy) octadecanoic acid, *EHOTT* 18-(4-ethylhexyloxy)-18-oxooctadecane-7,9,10-triyl tristearate.

### Tribological measurements

Lubrication is a process employed to reduce the wear of one or both surfaces in close proximity, and moving relative to each other, by interposing a substance called a lubricant between the surfaces to carry or to help carry the load (pressure generated) between the opposing surfaces. Adequate lubrication allows smooth continuous operation of the equipment with only mild wear and without excessive stresses at the bearings. When the lubricant breaks down, metal or other components can rub destructively over each other, causing damage, heat, and failure. Tribology is the interaction between surfaces in relative motion. The tribological interactions of a solid surface's exposed face with interfacing materials and the environment may result in loss of material from the surface.

The process leading to loss of material is known as “wear.” Major types of wear include abrasion, friction (adhesion and cohesion), erosion, and corrosion. Wear can be minimized by modifying the surface properties of solids by one or more “surface engineering” processes (also called surface finishing) or by the use of lubricants (for frictional or adhesive wear) [40]. A lubricant is any material that is interposed between surfaces, forming a film to avoid or minimize contact between the surfaces. The main functions of a lubricant are to reduce wear on components due to friction, to cool, to protect against corrosion and to clean [41]. An important property of lubricants is their ability to maintain a stable lubricating film at the metal contact zone. Plant oils and fatty esters are known to provide excellent lubricity due to their ester functionality. The ester ends of the fatty acid chain adsorb onto the metal surfaces, thus forming a monolayer film with the hydrocarbon end of the fatty acids oriented away from the metal surface [42]. The fatty acid chain thus offers a sliding surface to prevent direct metal-to-metal contact. If the film is not formed, direct metal contact may result in high temperatures at the zones of contact between the moving parts that cause adhesion, scuffing, or even metal-to-metal welding. The strength of a fluid film and its extent of adsorption onto the metal surface dictate the efficiency of a lubricant’s performance. In addition, the friction coefficient and wear rate are dependent on the adsorption energy of the lubricant [43,44].

Table 6 shows the tribological results for the synthesized compounds **2**–**5**. The results indicate that an improvement in the anti-wear/anti-friction properties (reduced wear scar diameter (WSD) values and coefficients of frictions (μ)) occurred with an increase in the number of substituents at the mid- and end-points. This result may be due to the increasing polarity in the structure with increasing numbes of ester functional groups, i.e., tetraester > triester > monoesters (Table 6), which will increase the strength of the tribological film at the metal contact zone and thereby increase its efficiency in reducing the wear/friction [45,46].

**Table 6:** The tribological properties of the samples in the four-ball test

**Samples**	**Four ball wear scar diameter (WSD), 40 daN, 1 h (mm)**	**Coefficient of friction (μ)**
**2**, ERA	1.14	0.75
**3**, OHSOA	0.91	0.69
**4**, TSOA	0.78	0.57
**5**, EHOTT	0.62	0.44

*ERA* epoxidized ricinoleic acid, *DHSOA* 10,12-dihydroxy-9-(stearoyloxy) octadecanoic acid, *TSOA* 9,10,12-tris(stearoyloxy) octadecanoic acid, *EHOTT* 18-(4-ethylhexyloxy)-18-oxooctadecane-7,9,10-triyl tristearate.

## Conclusions

The widespread use of plant oils as biolubricant basestocks will depend largely on how well they perform during high-temperature oxidation and low-temperature applications. Oxidative stability and low-temperature characteristics of plant oils should be improved before these oils are considered for universal biolubricant application. The complexity of plant oil oxidation is primarily due to the involvement of different structural parameters in the fatty acid chain. Different structural parameters participate in the reaction at different stages of oxidation. Based on the results obtained from this work, the chemical modification of ricinoleic acid led to decreased pour points because of, the increased ability to disrupt crystalline formation at reduced temperatures; increased viscosity index because of, the increased molecular weight of the synthesized compounds; increased onset temperature together with decreased volatile loss and insoluble deposits because of, elimination of the double bonds of ricinoleic acid; and decreased coefficients of friction because of, the increasing number of polar functional groups in the structures of the synthesized compounds. These changes led to stronger adsorption onto the metal surface and enhanced lateral interactions. For example, elimination of the double bonds together with attachment of mid- and end-chain ester groups generally led to improved physicochemical and tribological properties of the synthesized products.

## Experimental and methods

### Characterization

^1^H and ^13^C NMR spectra were recorded using a JEOL JNM-ECP 400 spectrometer operating at frequencies of 400.13 and 100.77 MHz, respectively, with a 5-mm broadband inverse Z-gradient probe in DMSO-d_6_ (Cambridge Isotope Laboratories, Andover, MA). Each spectrum was Fourier-transformed, phase-corrected and integrated using MestRe-C 2.3a (Magnetic Resonance Companion, Santiago de Compostela, Spain) software. The FTIR spectra were recorded directly on a Thermo Nicolet Nexus 470 FTIR system (Madison, WI) with a Smart ARK accessory containing a 45 ZeSe trough plate over a scanning range of 650–4,000 cm^–1^ using 32 scans to yield a spectral resolution of 4 cm^–1^.

### Pour point and cloud point

The pour points (PP) and cloud points (CP) were measured using the ASTM D5949 [47] and ASTM D5773 [48] methods, respectively, with a Phase Technology Analyzer, Model PSA-70S (Hammersmith Gate, Richmond, B.C., Canada). The pour point, or the temperature at which a lubricant ceases to flow, is important in appraising the flow properties at low temperature and, as such, can become the determining factor for selecting a lubricant. In addition, the pour point can be defined as the minimum temperature of a liquid (particularly a lubricant) below which the liquid ceases to flow and, along with pump-ability, as the ease with which the oil pumps at low temperatures, which is a significant factor in cold-weather start-up. The cloud point is the lowest temperature at which the sample becomes clouded by the formation of wax crystals. Clouding is only characteristic of paraffinic oils and is a consideration in the evaluation of fuels whose filtration might be impaired by the plugging effect of wax crystals. All of the runs were carried out at least twice, and the average values are reported.

### Viscosity index measurements

Automated multi-range viscometer tubes HV M472 obtained from Walter Herzog (Germany) were used to measure the viscosity. The measurements were made in a Temp-Trol (Precision Scientific, Chicago, IL, USA) viscometer bath set at 40.0 or 100.0°C. The viscosity and the viscosity index were calculated using ASTM methods D445-97 [49] and D2270-93 [50], respectively. All the measurements were made in triplicate, and the average values are reported.

### Pressurized differential scanning calorimetry (PDSC) method

It is well known that when plant oils are exposed to an oxidizing environment, they undergo oxidative degradation. Oxidation is the single most important reaction of oils used as lubricant base oils, resulting in increased acidity, corrosion, viscosity, and volatility. Therefore, understanding and controlling oxidation is a major concern for lubricant chemists. A primary tool employed to determine the oxidation of lubricants is differential scanning calorimetry (DSC) or pressurized differential scanning calorimetry (PDSC), where the oxygen concentration is adjusted to exceed that at ambient pressure to expedite the oxidation process. The measurements using pressurized differential scanning calorimetry offer two main benefits. First, the use of high pressure helps to decrease the sample volatility by elevating the boiling points. Second, high pressure increases the concentration of the reacting gases, which allows the use of lower test temperatures or shorter test times at the same temperatures. The remaining useful life of lubricants evaluated using a pressurized differential scanning calorimetry technique was shown to be more accurate than the results obtained using differential scanning calorimetry. In this work, the oxidative stability experiments were performed using a DSC 2910 thermal analyzer from TA Instruments (New Castle, DE). Typically, a 1.5-2.0 mg sample was placed in a hermetically sealed aluminum pan with a pinhole lid for interaction between the sample and the reactant gas (dry air). A film thickness of less than 1 mm was required to ensure proper oil–air interaction and to eliminate any gas-diffusion issues. The dry air (Gateway Airgas, St Louis, MO) was pressurized in the module at a constant pressure of 1379 kPa. A 10°C/min heating rate was used to raise the temperature of the materials from 50 to 350°C during each experiment. The onset temperature (OT, °C) and the signal maximum temperature (SMT, °C) of the oxidation were calculated from the exothermal reaction of each sample. Each test was run in triplicate, and the average values are reported.

### The thin-film micro-oxidation (TFMO) method

The thin-film micro-oxidation test is often the method of choice for studying plant oils’ thermal-oxidative stability because it is simple and reproducible. The test oil (25 μL) was spread as a thin film on a freshly polished high-carbon steel catalyst surface and was oxidized by passing a steady flow (20 cm^3^/min) of dry air over the heated sample. The oxidation was carried out at a constant temperature (175°C) inside a glass-bottomed reactor. The temperature was maintained at ±1°C by the placement of a heated aluminum slab atop a hot plate. This arrangement eliminated the temperature gradient across the aluminum surface and transferred the heat to the catalysts placed on the slab. The constant air flow ensured the removal of volatile oxidation products. The test was designed to eliminate any gas diffusion limitations. After a specific time, the catalyst and the oxidized oil sample were removed from the oxidation chamber, rapidly cooled under a steady flow of dry N_2_ and immediately transferred to desiccators for temperature equilibration. After approximately 1 h, the catalyst containing the oxidized oil was weighed to determine the loss of volatile compounds due to thermal evaporation or the gain of material due to oxidation. The sample was then soaked in tetrahydrofuran (THF) for 30 min to dissolve the soluble portion of the oxidized oil. After dissolving the soluble oil, the catalyst containing the insoluble portion was placed into a desiccator to remove the last traces of the solvent. The sample was then weighed to determine the mass of the insoluble deposit. Each test was run in triplicate, and the average values are reported.

### The density determination method

The densities were determined at 20°C based on the ASTM method D1298-99 [51] using a glass hydrometer provided by Lanxi Comp., Shanghai, China.

### The volatility determination method

The volatility was determined in agreement with ASTM method D6184 [52] in an electric stove using glass pans of 4 cm in diameter.

### The tribological test method

The experiment is designed to study the anti-wear properties under sliding contact by four-ball test geometry. The test zone is a top ball rotating in the cavity of three identical balls in contact and clamped in a cup below, containing the test fluid. The resistance to the motion of the ball is measured by a load cell connected to the stationary cup on the load platform, containing the 3 balls. Appropriate load is applied from below and the top ball is rotated at a set speed for a particular length of time. The balls were thoroughly cleaned with methylene chloride and hexane before each experiment. Test fluid (10 ml) was poured in the test cup to cover the stationary ball. The test sequence allowed the speed to attain a set rpm of 1200 before a normal load of 40 Kg was applied at room temperature for 15 min. Temperature of the test fluid was 22°C which increased to 27–28°C at the end of the 15 min run. In this work, the tribological determinations were performed according to ASTM method D4172-94 [53] using the Anton Paar Physica MCR301 apparatus (Germany) with Rheoplus/32 V3.40 software. Each test was run in triplicate, and the average values are reported.

## Materials

Ricinoleic acid (99%), formic acid (88%) and hydrogen peroxide (30% solution) were obtained from ChemR (Poland). Stearic acid, *p*-toluenesulfonic acid (PTSA) and toluene where obtained from Fisher Scientific. Sulfuric acid and 4-ethylhexanol were obtained from Merck. Hexane was obtained from Aldrich. All other chemicals and reagents were obtained from Aldrich Chemical (Milwaukee, WI). All materials were used without further purification. All organic extracted were dried using anhydrous magnesium sulfate.

### Synthesis

#### Epoxidized ricinoleic acid (ERA) (2)

A solution of hydrogen peroxide (30% in H_2_O, 8.0 mL) was slowly added to a stirred solution of ricinoleic acid (RA) **1** (95%, 15 g) dissolved in formic acid (88%, 14 mL) at 4°C (ice bath). The reaction proceeded at room temperature with vigorous stirring (900 rpm) until a powdery solid formed in the reaction vessel (2–5 h). The solid was collected via vacuum filtration, washed with H_2_O (chilled, 3 × 10 mL) and dried for 12 h under high vacuum to provide epoxidized ricinoleic acid as a white, powdery solid (14.7 g, 93%).

#### Synthesis of 10,12-dihydroxy-9-(stearoyloxy) octadecanoic acid (DHSOA) (3)

Stearic acid (12 g) was slowly added to a mixture of epoxidized ricinoleic acid **2** (31 g) and *p*-toluenesulfonic acid (PTSA) (5 g) in toluene over 1.5 h, and the temperature of the reaction mixture was kept at 70–80°C. The reaction mixture was subsequently heated to 90–100°C and refluxed for 3 h. After the reaction was complete, the solution was allowed to cool to room temperature and stirred overnight. The next day, the mixture was washed with water, the organic layer was dried over anhydrous magnesium sulfate and the solvent was removed using a vacuum evaporator.

#### Synthesis of 9,10,12-tris(stearoyloxy) octadecanoic acid (TSOA) (4)

Sulfuric acid (conc. H_2_SO_4_, 10 mol-%) was added to a stirred suspension of 10,12-dihydroxy-9-(stearoyloxy) octadecanoic acid **3** (3.35 mmol), and then stearic acid (24 g) was added to the reaction mixture. The suspension was stirred and heated at 60°C for 10 h. Next, hexane (5 mL) was added, and the solution was washed once with saturated aqueous NaHCO_3_ (0.5 mL) and brine (2 × 1 mL), dried (MgSO_4_), filtered, and concentrated under vacuum for 6 h to yield the target product.

#### Synthesis of 18-(4-ethylhexyloxy)-18-oxooctadecane-7,9,10-triyl tristearate (EHOTT) (5)

The reaction scheme for the formation of the diesters is provided in Figure 1. Sulfuric acid (conc. H_2_SO_4_, 10 mol-%) was added to a stirred suspension of 9,10,12-tris(stearoyloxy) octadecanoic acid **4** (3.35 mmol) in 4-ethylhexanol (3.35 mL). The suspension was stirred and heated to 60°C for 2 h. Next, hexane (5 mL) was added, and the solution was washed once each with saturated aqueous NaHCO_3_ (0.5 mL) and brine (2 × 1 mL), dried (MgSO_4_), filtered, and concentrated under vacuum for 6 h to yield the target product. The molecular weight was determined using LC-MS (ToF) Bruker Delton q-ToF. The molecular weight determined was 1244.07.

## Competing interests

The authors declare that they have no competing interests.

## Authors’ contributions

NS and JS developed the concept analyzed the data and drafted the manuscript. EY provided advice on the testing methods. BMA performed the characterization methods. All of the authors read and approved the final manuscript.
